# Oligodendrocyte differentiation of induced pluripotent stem cells derived from subjects with schizophrenias implicate abnormalities in development

**DOI:** 10.1038/s41398-018-0284-6

**Published:** 2018-10-23

**Authors:** Donna L. McPhie, Ralda Nehme, Caitlin Ravichandran, Suzann M. Babb, Sulagna Dia Ghosh, Alexandra Staskus, Amy Kalinowski, Rupinderjit Kaur, Panagiotis Douvaras, Fei Du, Dost Ongur, Valentina Fossati, Kevin Eggan, Bruce M. Cohen

**Affiliations:** 1 Harvard Medical School, Boston, MA USA; 2 McLean Hospital, 115 Mill St., Belmont, MA 02478 USA; 3 Stanley Center for Psychiatric Research, Broad Institute of Harvard and MIT, Cambridge, MA 02142 USA; 4 Department of Stem Cell and Regenerative Biology, Harvard University, Cambridge, MA 02138 USA; 5 Blue Rock Therapeutics, Alexandria Center for Life Science, 450 E 29th Street, Suite 504, New York, NY 10016 USA; 6 The New York Stem Cell Foundation Research Institute, 619 West 54th Street, 3rd Floor, New York, NY 10019 USA; 7 Stanley Center for Psychiatric Research and Program in Medical and Population Genetics, Broad Institute of Harvard and MIT, Cambridge, MA 02142 USA

## Abstract

Abnormalities of brain connectivity and signal transduction are consistently observed in individuals with schizophrenias (SZ). Underlying these anomalies, convergent in vivo, post mortem, and genomic evidence suggest abnormal oligodendrocyte (OL) development and function and lower myelination in SZ. Our primary hypothesis was that there would be abnormalities in the number of induced pluripotent stem (iPS) cell-derived OLs from subjects with SZ. Our secondary hypothesis was that these in vitro abnormalities would correlate with measures of white matter (WM) integrity and myelination in the same subjects in vivo, estimated from magnetic resonance imaging. Six healthy control (HC) and six SZ iPS cell lines, derived from skin fibroblasts from well-characterized subjects, were differentiated into OLs. FACS analysis of the oligodendrocyte-specific surface, glycoprotein O4, was performed at three time points of development (days 65, 75, and 85) to quantify the number of late oligodendrocyte progenitor cells (OPCs) and OLs in each line. Significantly fewer O4-positive cells developed from SZ versus HC lines (95% CI 1.0: 8.6, F_1,10_ = 8.06, *p* = 0.02). The difference was greater when corrected for age (95% CI 5.4:10.4, F_1,8_ = 53.6, *p* < 0.001). A correlation between myelin content in WM in vivo, estimated by magnetization transfer ratio (MTR) and number of O4-positive cells in vitro was also observed across all time points (F_1,9_ = 4.3, *p* = 0.07), reaching significance for mature OLs at day 85 in culture (*r* = 0.70, *p* < 0.02). Low production of OPCs may be a contributing mechanism underlying WM reduction in SZ.

## Introduction

Schizophrenia (SZ) is a complexly determined neurodevelopmental disorder affecting approximately 1% of the population and often creating lifelong disability. The expression of SZ depends on interactions among thousands of genes and environmental factors. Because of the large number of causal factors, individual cases may have unique elements of etiology. However, at the level of clinical presentation, there are common, even stereotypical, features. Some altered pathways of brain development appear to be shared across cases of SZ, producing the syndromic outcome. Among these pathways, there is consistent evidence of abnormalities of brain connectivity and signal transduction in individuals with SZ^1,2^. Relevant to those findings and to the studies reported here, there is consistent and convergent evidence of abnormal myelination of neurons in the brain in SZ^3^.

The evidence of myelination anomalies in SZ arises from a growing number of studies, using diverse technologies including brain imaging, post-mortem (PM) tissue analyses, gene-set (pathway) analyses, genome wide association studies (GWAS), and gene expression studies. All implicate abnormalities of myelin levels or myelination and of OLs, the cells that produce myelin^4–8^.

In vivo, magnetic resonance (MR) diffusion tensor spectroscopy studies point to reduced and disorganized WM in psychotic disorders, especially in SZs^2,9^. Utilizing magnetization transfer ratio (MTR) techniques in vivo, Du et al.^2^ observed a reduction in a measure directly associated with myelin in subjects with SZ when compared to healthy controls (HC). They also found an elevation of the diffusion coefficient of the intraneuronal molecule *N*-acetylaspartate in SZ, which may reflect a widening of axon bores to compensate for reduced myelination^10^. A number of PM studies found a reduction in WM in the prefrontal cortex, an area critical in higher order processing of information and known to be affected in psychosis^11–14^. Studies of gene regulation in PM brain report reduced expression of genes related to OL development and myelin production^5,15^. Additionally, a recent transplantation study by Windrem et al.^8^ provided confirmatory evidence of pathology of glial cells derived from subjects with SZ. The authors created chimeric mice by injecting iPS cell-derived glial precursor cells (GPCs) from subjects with familial childhood onset SZ and age-matched controls into a hypomyelinated mouse model. The SZ GPCs injected into mice showed abnormalities in migration and produced general hypomyelination, as well as neurophysiologic abnormalities, compared to controls.

In a large GWAS study, 108 genetic loci were associated with SZ to a statistically significant degree^16^. However, no variants explained much of the risk, and it was estimated that upwards of 8000 loci may contribute to risk of illness. Given that the interactions of numerous genes determine risk, one way to increase signal from the GWAS data is to look at the association of gene sets, rather than single genes, with illness, using pathway analysis. Applying these techniques, studies by our group^7^ and others^17^ found strong associations of gene sets for the development and function of OLs and SZ.

The complex determinants of SZ, involving cell growth and function, are difficult to study in vivo, due to limited access to brain. MR studies only determine bulk characteristics of tissue, limiting the ability to relate brain abnormalities to underlying molecular and cellular determinants and processes. Animal models cannot necessarily replicate the phenomena of SZ. In particular, animal models are not optimal for the study of glial cell development, because the pathways determining glial development and function in rodents appear to be fundamentally different in crucial ways from those in humans^18^.

Human cell models in culture might address some of these problems, and they offer a view of disease related processes at a functional level complementary to genomics and brain imaging studies. SZ is a developmental disease, and anomalies appear early, even before the overt onset of symptoms. Reprogrammed cells offer a good opportunity to study and monitor the development of specific cell types from precursors through mature differentiation. The results can be combined with findings from brain MR and genomic studies to define specific mechanisms and pathways of abnormal development and function and, eventually, associations with clinical phenotype.

Direct characterization of the role of OL dysfunction in psychotic disorders, and more specifically SZ, on the cellular level is now possible because of advances in the derivation of adult stem cells and specific neural cell differentiation paradigms in vitro. Developed over the last ten years, these techniques allow investigation of specific differences in neural and glial development in well-controlled cell culture experiments^19–22^.

We performed studies to determine the potential of in vitro OL generation in cell lines derived from iPS cells from subjects with SZ and HC. The studies were designed to determine whether OLs from SZ were abnormal in culture and whether the abnormalities observed were concordant with anomalies seen by in vivo brain MR studies. OL development was compared with in vivo measures of WM integrity and myelination in the same subjects who donated the cells for the generation of iPS lines. The results directly confirm the presence of OL abnormalities in SZ, as suggested by previous studies, advance knowledge on the specific nature of OL dysfunction, and further suggest that the behavior of cells in culture may be correlated with the characteristics of these human cells in whole living brain.

## Methods

Research subjects: Subjects included six HC and six patients with SZs, including schizoaffective disorder (together: SZ), recruited from the clinical services at McLean Hospital. Participants were assessed using the Structured Clinical Interview for the DSM-V (SCID)^23^ and did not have significant acute or substantial chronic medical illness or conditions. Subjects were studied for brain myelin content in a previous protocol^2^. Detailed inclusion and exclusion criteria are given in that reference. There are no previous data on the degree of oligodendroglia production abnormalities in SZ, but sample size was chosen on the basis of the myelin and WM abnormalities previously reported^2^. Our prior expectation was that we would be able to see differences as great as those observed on brain imaging. Subject characteristics are described in Table 1.

**Table 1:** Subject characteristics

	Healthy controls (*n* = 6)	Schizophrenias (*n* = 6)
SCID-V Diagnosis	6 HC	4 SZ/2 SZA
Sex	5 M/1 F	4 M/2 F
Mean age (years) at skin biopsy	29.7 ± 9.9 years	39.7 ± 4.8 years
Age range at skin biopsy	21–45	33–45
Mean age (years) at MR scan	30.5 ± 10.3 years	40.0 ± 6.1 years

All protocols were approved by the Partners Healthcare Institutional Review Board, and subjects provided written informed consents before participation in any studies.

### Derivation and expansion of fibroblasts

Fibroblasts were grown from 3 mm skin biopsies, cut into small pieces, placed in plate wells with 2 ml minimal essential media plus 15% fetal bovine serum and 1% Penn/Strep under coverslips for 7 days in an incubator at 37 °C, 5% CO^2^. Coverslips were removed when cells were clearly seen migrating out of tissue pieces. Cells were passaged when 80–95% confluent, first to one 100 mm dish, then to five 150 mm dishes, then frozen, and stored. Fibroblast stocks were tested for mycoplasma.

### Conversion to induced pluripotent stem cells

Fibroblasts were sent to either the New York Stem Cell Foundation Research Institute (NYSCF) or Cellular Reprogramming, Inc. (San Diego, CA) for RNA reprogramming. Four SZ and five HC lines were derived at NYSCF and two SZ and one HC lines (converted as part of another study) were from Cellular Reprogramming. NYSCF reprogramming is based on mRNA/miRNA transfection as detailed in Paull et al.^24^. Fibroblasts sent to Cellular Reprogramming were similarly converted and the resulting iPS cell colonies were stabilized and expanded. Each fibroblast line was plated to six-well plates without feeders at three different plating densities and subjected to messenger RNA reprogramming. Colonies were bulk-passaged from the most productive well to establish passage 1 iPS cell cultures on rLaminin-521 (BioLamina) in Nutristem XF media (Biological Industries) and expanded in the same culture system until at least passage 3 before being characterized by DAPI/OCT4/TRA-1-60 immunostaining and frozen down. Cells from both sources were made with the same technology and had similar properties.

### Characterization of iPSC

After conversion, iPSC lines from NYSCF were characterized as in Paull et al.^24^. Briefly, Nanostring analysis was performed using a 25 gene probe set for markers of pluripotency. Differentiation potential into the three germ layers was assessed using an expression panel of 100 gene probes, after spontaneous differentiation of iPSCs into embryoid bodies. Additionally, cells were karyotyped with the Nanostring nCounter Plex2 Assay Kit. iPSCs reprogrammed at Cellular Reprogramming showed human embryonic stem cell-like morphology and expressed human pluripotent stem cell markers. Characterized iPSCs from both sources were returned to McLean for subsequent studies. iPSCs were mycoplasma tested prior to plating for differentiation.

### OL differentiation

The 12 subject lines of iPSCs were differentiated three times to OLs as in Douvaras and Fossati^25^ following the fast version of the differentiation protocol. A Scepter 2.0 automated cell counter (Millipore Sigma) was used for all cell counts.

### Excitatory neuron differentiation

iPSCs were differentiated into excitatory neurons (hpiNs, human patterned induced neurons) and harvested after 28 days in culture as previously described^26^.

### Single cell RNA sequence characterization of OLs at D65

Plated neurosphere cultures were enzymatically harvested at D65 by Accutase treatment for 1 h at 37 °C; DNAse 1 was added for 5 min to digest genomic DNA released from ruptured cells. The cells were then passed through a 70 μm filter to obtain a single cell suspension. After pelleting, cells were re-suspended in 100 μl of their growth medium containing the appropriate amount of O4-488 conjugated antibody. Stained cells were then pelleted and washed with phosphate-buffered saline and sorted into a 96-well plate using a BD Biosciences FACSAria III, 100μm ceramic nozzle, on the slow setting. SmartSeq2 libraries were prepared according to the SmartSeq2 protocol^27,28^.

Sequencing was carried out as paired-end 2 × 25 bp with an additional eight cycles for each index. Data were separated by barcode and aligned using Tophat version 2.0.10 (ref. ^29^) with default settings. Transcripts were quantified using the Cufflinks suite version 2.2.1 (ref. ^30^). Data were normalized for gene length and library size using Cuffnorm.

### Immunofluorescent characterization of the O4-expressing OLs

Primary antibody anti-O4 or anti-O1 diluted (1:100) and 5% goat serum was added to live cultures and incubated for 45 min at 37 °C. After one wash with pre-warmed media, secondary goat anti-mouse Alexa Fluor-488 or Alexa Fluor-568 was added for 30 min at 37 °C. Cultures were washed and fixed with 4% paraformaldehyde in 100 mM phosphate buffer pH 7.4. All other immunocytochemistry was done post fixation. Primary antibodies were used at the following dilutions: rabbit anti-OLIG2 (1:500), goat anti-SOX10 (1:100), and rat anti-MBP (1:200). Secondary antibodies, donkey or goat anti- rabbit, mouse, goat or rat Alexa Fluor-488, 555, or 568 were used at 1:500. DAPI staining was used to visualize cell nuclei in ICC protocols. Antibody manufacturer, catalog number, and dilution information are listed in Table 2.

**Table 2:** List of antibodies used

Antibody	Manufacturer	Catalog #	Dilution
Anti-O4 (raised in mouse)	R&D Systems	MAB1326	1:100
Anti-O1 (raised in mouse)	R&D Systems	MAB1327	1:100
OLIG2 (raised in rabbit)	EMD Millipore	AB9610	1:500
SOX10 (raised in goat)	R&D Systems	AF2864	1:100
MBP (raised in rat)	Abcam	Ab7349	1:200
O4-488 (directly conjugated)	R&D Systems	FAB1326G	1:100
Goat anti-mouse Alexa Fluor-488	Thermo-Fisher	A-11029	1:500
Goat anti-mouse Alexa Fluor-568	Thermo-Fisher	A-11004	1:500
Goat anti rat Alexa Fluor-488	Thermo-Fisher	A-11006	1:500
Goat anti rabbit Alexa Fluor 555	Thermo-Fisher	A-21428	1:500
Donkey anti goat Alexa Fluor-568	Thermo-Fisher	A-11057	1:500

### Flow cytometry analysis of O4-expressing cells

Plated neurosphere cultures were enzymatically harvested as detailed above at designated developmental time points. Sorting was done on a BD Biosciences Accuri C6 Cell Sorter using a 100 μm ceramic nozzle, on the slow setting. Fibroblasts that did not express O4 were used as a biological control for gating. Propidium iodine (PI) was used to identify and exclude dead cells. Data obtained were analyzed using BD C6 analysis™ software.

### Flow cytometry analysis of live and dead cells

Analysis of the percentage of live cells in mixed neurosphere cultures was done by FACS analysis according to the manufacturer’s protocols using the LIVE/DEAD Viability Cytotoxicity Kit, for mammalian cells.

### Magnetization transfer ratio measurements

All subjects participating in the study had MR imaging done and MTR data collection was as described in previous publications^2,31^. Data were collected from a 1 × 3 × 3 cm voxel (Fig. 4a) within the WM of the right prefrontal cortex at 4 Tesla (Varian/UnityInova; Varian Inc., Palo Alto, CA).

### Brain WM quantification

At 4 T high-contrast T1-weighted axial and sagittal images were also collected (TE/repetition time = 6.2/11.4 ms, field of view = 22 × 22 × 16 cm^3^, readout duration = 4 ms, receive bandwidth = ± 32 kHz, matrix size = 256 × 256 × 64, in-plane resolution = 0.94 × 0.94, slice thickness = 2.5 mm, flip angle = 11°). Whole-brain images were segmented into gray matter, WM, and CSF using FSL’s FS-FAST^32^ routine and percent WM was calculated.

### Data analysis

Laboratory staff who isolated, reprogrammed, grew, and analyzed cells in vitro were blind to subject diagnosis and to the in vivo brain imaging results. The statistician (CR) was given access to all these data for the purpose of analysis.

Repeated measures linear regression tests were performed to detect associations of diagnosis, MTR, and percentage WM with percentage OLs in culture. Covariates were diagnosis (SZ patient or HC) and time in culture (in categories: days 65, 75, or 85 of differentiation). O4 percentages for the 3-day 85 replicates were averaged to obtain a single day 85 O4 percentage. We first assessed for statistical evidence of a difference in diagnosis-associated reduction in percentage OLs between time points (a test for interaction between diagnosis and time). In the absence of statistical evidence of that interaction, we tested for evidence of the primary hypothesis, a patient–control difference in percentage OLs in culture in a model assuming the same or similar difference across time points. To test the secondary hypothesis, whether there was an association between MTR or percentage WM (as quantified by FSL-FMRIB Software Laboratory v5.0) in vivo with percentage OLs in vitro, we replaced diagnosis with either brain myelin content (MTR) or percentage brain WM as a covariate in the model. We allowed unstructured covariance between repeated measurements, and used Kenward–Roger degrees of freedom for hypothesis testing. A sensitivity analysis tested for an association of diagnosis with percentage OLs, controlling for age. Finally, Pearson correlations were used to quantify associations between continuous measures.

### Analysis of CSPG4 variants

Exome sequencing was done as part of a larger effort at the Stanley Center at the Broad Institute. Variants of CSPG4 were analyzed using the Integrative Genomics Viewer^33,34^.

## Results

### Derivation of OLs

In order to investigate whether low myelination levels and abnormalities in OLs seen in SZ in vivo would be associated with lower numbers of OPCs and OLs derived from subjects iPS cells in vitro, OLs were differentiated from iPS cells from six SZ and six HC subjects, three independent times, as previously described^25^.

**Fig. 1:**
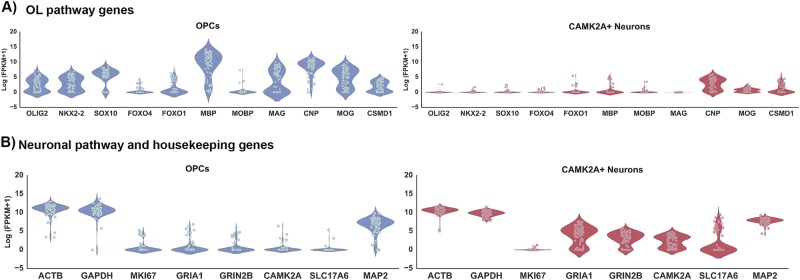
Single cell characterization by RNA expression of OL and neuron-related genes in derived OPCs and CAMK2 + neurons. **a** At the D65 time point of OL differentiation, several specific OL genes, specifically OLIG2, NKX2.2 SOX10, MBP, MAG, and MOG, were expressed in OPCs (blue) but not present in D28 CAMK2A + hpiNs (human patterned induced neurons) (red). **b** Expression profile for a set of neuronal genes in CAMK2A + hpiNs harvested at day 28 post-induction shows distinct enrichment for neuron-specific gene expression. D65 OPCs do not show specific enrichment for neuronal genes. Housekeeping genes ACTB, GAPDH, and MAP2 show expression in each cell type. The proliferation marker MKI67 shows low to minimal expression in both cell types indicating maturity of the cells

**Fig. 2:**
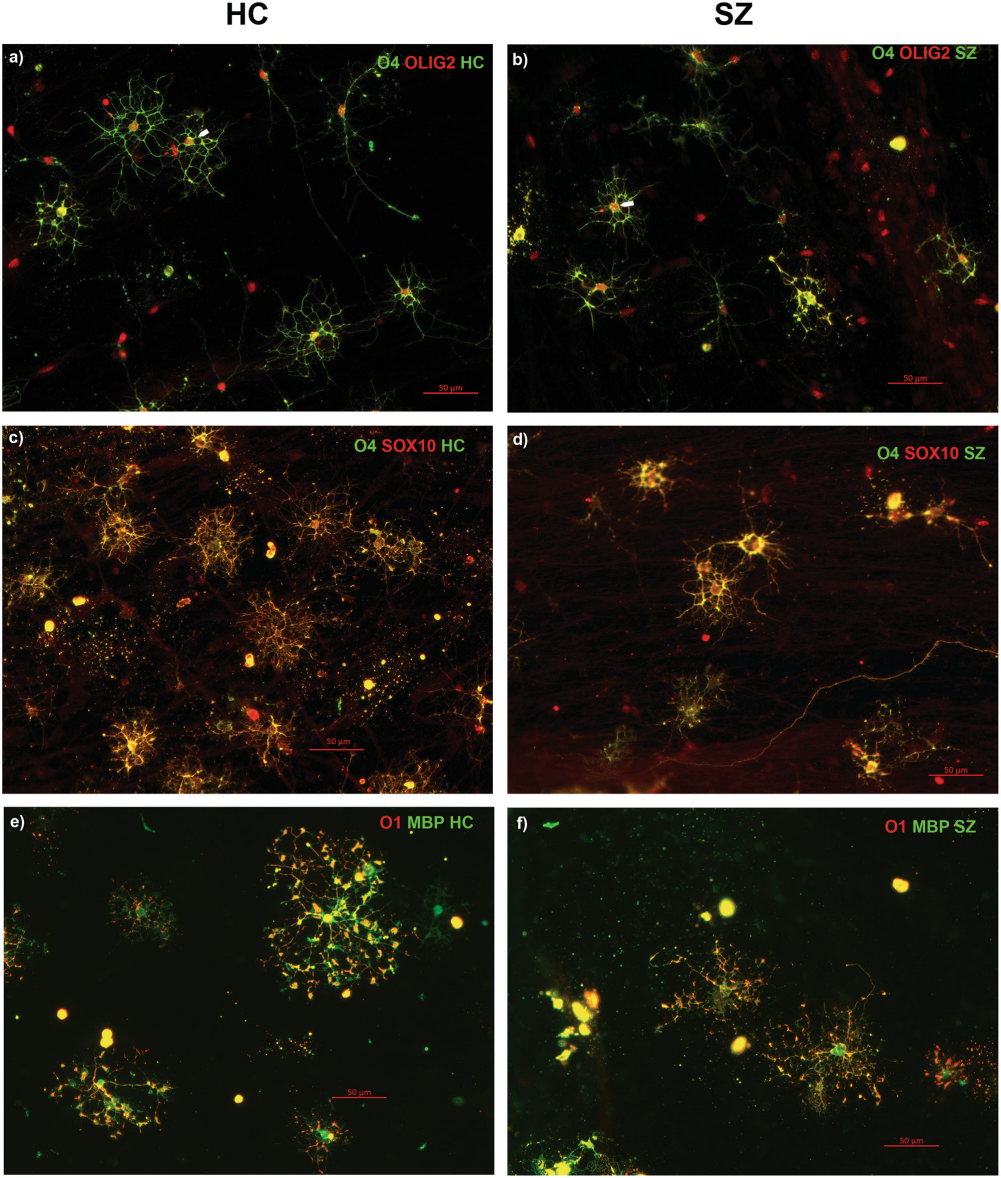
Immunofluorescent characterization of co-expression of OL pathway-specific markers. To further determine whether the O4-positive cells were co-expressing other OL-specific markers (OLIG2, SOX10, MBP, and O1) on the protein level, immunocytochemistry was performed for these protein markers, and co-expression was confirmed both in HC and SZ samples. Co-expression of OLIG2 (red) and O4 (green) in the same cells at differentiation D56 in HC (**a**) and SZ (**b**) samples. White arrows show examples of red OLIG2-positive nuclei in green O4-positive cells. SOX10 (red) and O4 (green) co-expression is shown at D65 in HC (**c**) and SZ (**d**). Myelin Basic protein (MBP) (green) and O1 (red) are seen at D85 in HC (**e**) and SZ (**f**). Note that yellow fluorescence indicates co-expression of the respective markers where both markers are predominantly in cytoplasm, as in panels **c**–**f**

Single cell total RNA sequence analysis was performed at D65 of the differentiation on one control subject line in order to verify that the derived cells were expressing markers characteristic of OL and not neuronal lineage, methods used as detailed in Picelli et al.^27,28^. The expression data from derived OPCs at D65 were also compared to D28 CAMK2A + hpiNs (human patterned induced neurons)^26^. Enhanced expression of OL lineage genes and minimal expression of neuron-specific genes were seen in derived OPCs (Fig. 1). Immunofluorescent staining for the co-expression of four key OL-specific markers was also performed in both HC and SZ samples to further confirm the identity of the O4-positive cells (Fig. 2a–d). In addition, O1 ICC at D85 confirmed the presence of mature OLs and the results showed that these mature OLs co-expressed MBP (Fig. 2e, f). The O1 antibody binds to galactocerebroside (GalC)^35^, a known marker of OL maturity.

### Testing the primary hypothesis: FACS analysis reveals SZ lines produce fewer OLs than controls

**Fig. 3:**
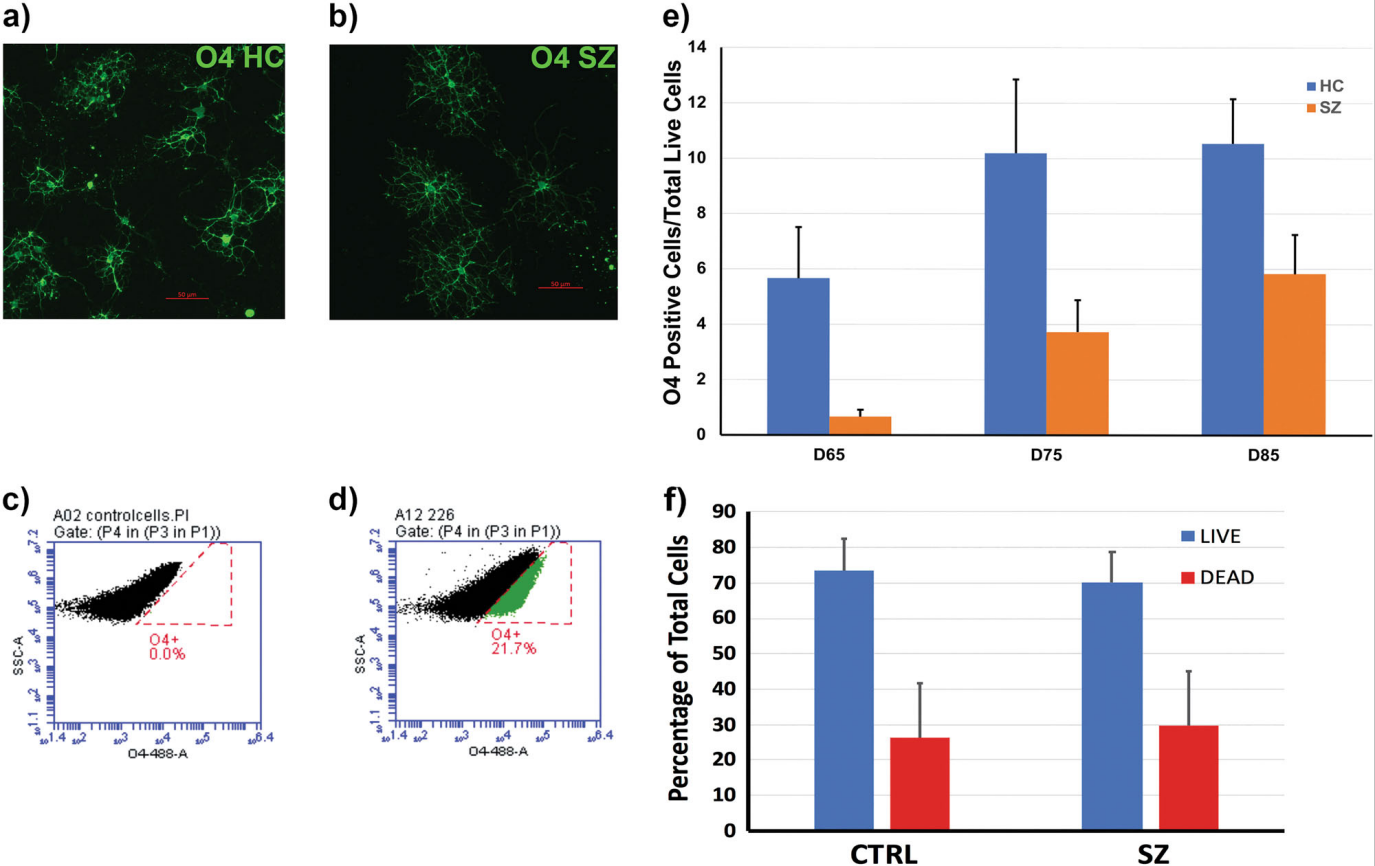
OL percentage by diagnostic group. An example of an immunofluorescent image of O4-positive cells from an HC (**a**) and an SZ (**b**) subject at D85. FACS profile analyses are shown for fibroblasts (**c**), as a biological control, and OPC/OLs (**d**), each incubated with O4-488 antibody. In **c,** **d** the Side Scatter Light (SSC-A) axis shows the percentage of cells that are alive and, therefore, propidium iodine (PI) negative. Earlier gates were used to eliminate cells under a size limit, doublets, and to exclude PI-positive dead cells. Black indicates the cell population is live and O4-488 negative; green indicates the cell population is live and O4-488 positive. Cell lines from the SZ group produced significantly fewer OLs than those from the HC group at all time points: Graph **e** shows mean percentages of O4-positive cells by group; error bars are SD. Significant differences were seen between groups across time points (95% CI 1.0: 8.6, F_1,10_ = 8.06, *p* = 0.02). Graph **f** shows mean percentages and SD of live and dead cells in the two experimental groups from the live–dead flow cytometric analysis. No significant differences were seen between the groups for the relative percentages of live and dead cells

In order to directly characterize and quantify the number of O4-positive OLs produced by each of the six SZ and six HC lines during differentiation, we performed FACS analysis at three developmental time points; D65, D75, and D85. OLs at D85 are considered mature OLs in culture, confirmed by expression of the late markers O1 and MBP (Fig. 2e, f and ref. ^25^). There was a significantly lower number of O4-positive OLs present in the SZ group when compared to lines from HC subjects across time points (see Fig. 3). There was no significant interaction between diagnosis and time in culture (F_2,9_ = 0.36, *p* = 0.71). In the model assuming a constant difference between SZ and HC over time, as explained in Methods, diagnosis of SZ was associated with a statistically significant reduction in the proportion of OLs to other cells, specifically, from 10.68% in HC to 5.86% in SZ (95% CI 1.0: 8.6, F_1,10_ = 8.06, *p* = 0.02). This difference of 4.8 percentage points reflects a 50% absolute reduction of the number of OLs produced by the SZ versus the HC group. This association was stronger after adjusting for age (7.9 percentage point reduction in OLs/total cells counted, 95% CI 5.4:10.4, F_1,8_ = 53.6, *p* < 0.001).

### Flow cytometry analysis of live and dead cells

FACS analysis of the cell lines, using the LIVE/DEAD Viability Cytotoxicity Kit, at the terminal developmental time point of D85 showed no group-specific differences in the percentage of live and dead cells in the cultures (Fig. 3f). This suggests that a lack of production of O4-positive OLs may be the factor determining low cell percentage, while a higher number of dead cells would have suggested non-viability in the SZ condition.

### Exploratory analysis: relationship of in vitro differentiation to in vivo brain myelin as estimated by MTR and WM measures

In vivo MR brain imaging was performed on all 12 subjects from whom iPS cells were derived. MTR measurements were obtained on all but one subject (described below) and WM measurements were obtained on all subjects.

**Fig. 4:**
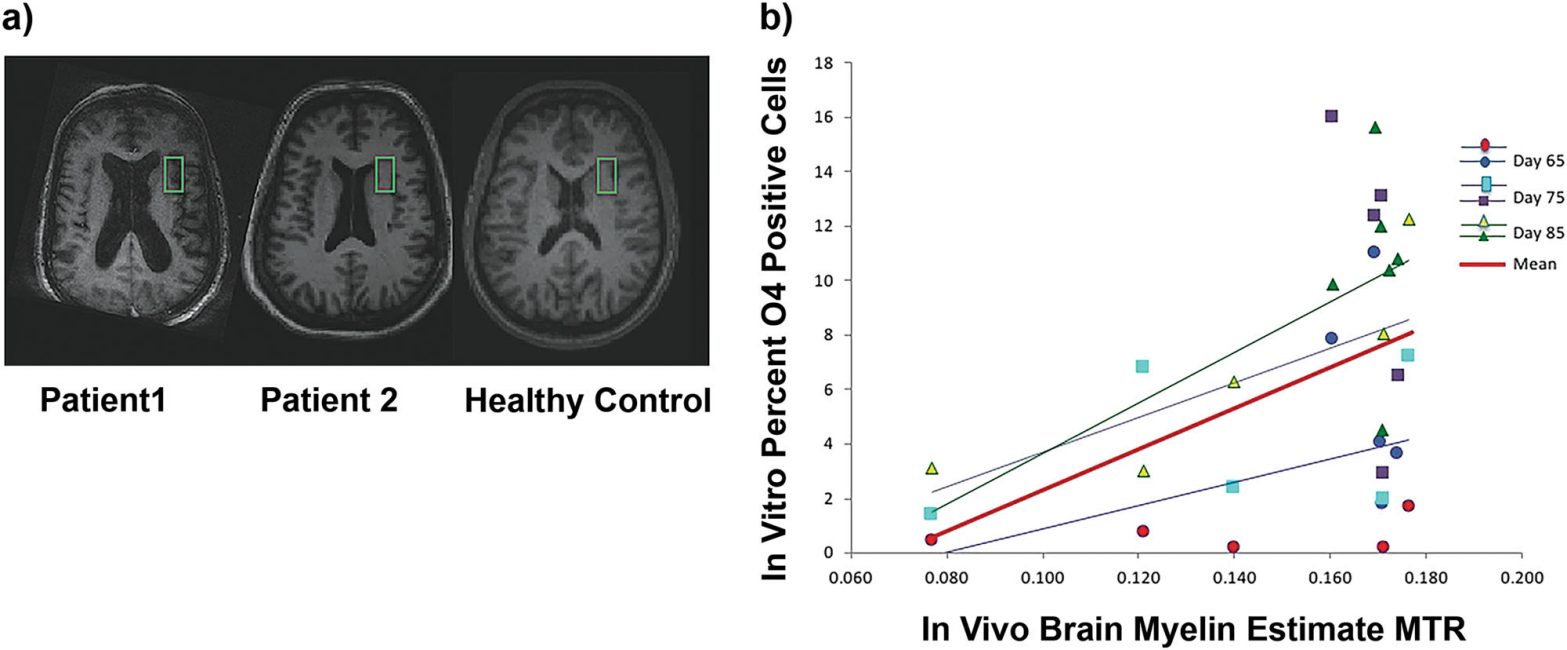
Correlation of O4 percentage in vitro to MTR (myelin estimate) in vivo. Illustrative axial T1-weighted MRI structural brain images. **a** The voxel used in the MTR measurements is shown in green. One subject with SZ (Patient 1) could not complete scanning to obtain MTR or quantitative WM measures, but we include his image to show an apparent low WM content. The next two images show subjects who completed MR scanning: Patient 2 (with SZ) and an HC subject. Correlation of percent O4-positive cells in vitro to MTR (myelin estimate) in vivo (b). Points represent each subject’s percent O4-positive cells versus in vivo brain myelin estimate using MTR. Points are shown for MTR versus O4-positive cells in culture at each of the three developmental times measured, with ordinary least-squares regression lines plotted separately for each day of measurement. The “Combined” line in red shows the fit of the general linear model associating percent O4 with MTR (F_1,9_ = 4.3, *p* = 0.07) averaged over the three developmental times. At D65, HC = blue circles, SZ = red circles; at D75, HC = purple squares, SZ = blue squares; at D85 HC = green triangles, SZ = yellow triangles

The brain localization of the voxel used is shown in Fig. 4a. Increased MTR was associated with increased percentage OLs in culture, though the association did not reach statistical significance for all time points combined (F_1,9_ = 4.3, *p* = 0.07) (Fig. 4b). One SZ subject could not tolerate MR scanning long enough to produce reliable data for MTR analysis, as noted. However, brain imaging for that subject showed very low WM volume (Fig. 4a, patient 1), consistent with the low production of OLs in cultures of cells from that subject. Estimated correlations between average percentage OL in culture and MTR were 0.56 (*p* = 0.08) for the full sample, 0.76 (*p* = 0.14) for SZ, and −0.42 (0.41) for HC. It is interesting that at D85, a time point when the largest number of mature OLs are present, the correlation between O4-positive cells and MTR is greatest and does reach significance (*r* = 0.70, *p* < 0.02 full sample; *r* = 0.86, *p*−0.06 SZ; *r* = −0.02, *p* = 0.96 HC). Despite the difference in correlation estimates between SZ and HC, the difference in association between O4-positive cells and MTR between SZ and HC was not statistically significant (F_1,7_ = 2.42, *p* = 0.17, test for interaction).

Modest trends to correlation were observed between in vivo myelin, estimated by MTR, and OLs in vitro at day 65 (*r* = 0.37, *p* < 0.29) and day 75 (*r* = 0.39, *p* < 0.27) of differentiation. In the whole dataset, percentage WM, as quantified by FSL, was not significantly associated with percentage OLs, although there was a trend in that analysis, in the same direction as seen in the MTR analysis (F_1,10_ = 1.92, *p* = 0.20). Individual subject values for MTR and WM are shown in Table 3.

**Table 3:** In vivo myelin and white matter estimates by magnetic resonance (MR) imaging

Sex	Diagnosis	Myelin estimate	WM %
M	Control	0.160	0.3259
M	Control	0.169	0.3764
M	Control	0.170	0.3189
M	Control	0.172	0.3158
M	Control	0.174	0.3276
F	Control	0.170	0.3310
F	SZ	0.176	0.3096
M	SZ	0.121	0.3826
M	SZA	0.140	0.3669
M	SZ	No value^a^	0.3293
M	SZ	0.077	0.3407
F	SZA	0.170	0.3261

Myelin is estimated from magnetization transfer ratio (MTR) results, see Methods

WM % = white matter percent of brain tissue by 4 T MR T1-weighted imaging, see Methods

^a^Inadequate time in an MR scanner to estimate MTR, see text

## Discussion

The results suggest that there are specific abnormalities of OL development in reprogrammed cells from subjects with SZ. It is also promising that the in vitro findings in culture show a trend to correlation with brain MR findings in vivo/in situ, as this suggests the abnormalities of cells in culture may be the same or closely related to abnormalities seen in brain development in the whole organism. This cell model might be used to clarify one pathophysiologic mechanism, abnormal myelination, contributing to the risk and development of SZ further and might even provide a platform to test methods or agents for amelioration or repair.

Potential mechanisms responsible for decreased numbers of differentiated OPCs and OLs in the patient samples may be complex, and not all relevant factors are known. Nonetheless, a number of attractive targets for investigation already exist. Evidence from the literature has focused on both early and late differences in the OL pathway that are associated with SZ. One example of this is OLIG2, a transcription factor expressed early on in OL development. OLIG2 has been shown to have decreased expression in SZ PFC by laser capture micro-dissection studies from PM human brain^15^. The RNA-binding protein QK1 is another provocative target in the hunt for a potential mechanism. QK1 expression has been shown to be associated with SZ in humans^36,37^. QK1 is also implicated as an interesting nexus point in the pathway of OL differentiation and maturation. Studies have shown that it may control the point of OL differentiation because it directly participates in the developmental regulation of myelin-associated glycoprotein (MAG)^38^. Evidence from the literature also shows that QK1 can influence the expression of MBP and SOX10, two other key genes in the OL development pathway^37^. In time, underlying genomic and biochemical mechanisms that explain the altered development of OLs can be identified. However, knowledge of specific biochemical pathways is not required for drug screens to identify agents that reverse abnormalities in OLs development and myelination. Rather, the ability to work with these cells in vitro opens possibilities to test interventions, in addition to offering a platform for finding mechanistic associations among various gene sets, diverse processes, OPC development, and in vivo phenotypic observations in SZ.

While this manuscript was being prepared, a paper by de Vrij et al.^39^ was published documenting an association of rare variants of CSPG4 and OPC dysfunction in some cases of familial SZ. While such cases do not explain the development of SZ in most instances, they represent a compelling demonstration of the relationship of OPC development and function to risk of SZ. An analysis of CSPG4 variants in the subjects with SZ found that none of our subjects had these rare variants. Therefore, our findings expand the evidence on OL abnormalities in SZ to a demonstration of a crucial relationship between dysfunctional OPC and SZ, in general.

We are not suggesting that abnormalities of OL development/myelin explain more than a part of the pathophysiology of SZ. It is well documented that SZ is a disorder involving not only many genes, but a variety of pathways and processes^7,17^. Still, the OL/myelin pathway appears to be an important determinant of illness and may be a site targetable for treatment development. In addition, it may not be necessary to address all the subtle mechanisms of SZ to bring substantial relief.

Also, we are not suggesting that cell types other than OLs do not matter in the expression of SZ. Abnormalities of several neuronal types have been observed in SZ, and there may be abnormalities of other glia, as well. In fact, some of the genes and processes altering OL development may be shared with neurons or other glia and be responsible for the diffuse structural, cellular, and functional abnormalities seen in SZ. Nonetheless, given the key role of myelination in guiding axon development and maintaining neuronal function, and the convergent evidence of OL developmental dysfunction in SZ, it would be reasonable to consider that the abnormalities observed here in OLs are likely to be of considerable relevance to SZ.

Limitations of the current study include the following: Typical of iPS cell studies, at least at this point in history, our sample is of modest size, though it is larger than those of most other iPSC studies of SZ in the literature. A larger sample size may have the power to detect a significant relationship between in vitro and in vivo measures. There was an age difference between groups, related to the availability of samples, but myelin changes only modestly from 20 to 50 years of age in HC subjects^40^ much below the differences observed here in OL production, and these changes are even smaller in people with SZ^41^. Cell reprograming was performed at two sites, but the reprogramming methods were the same, as were characteristics of the lines produced, as noted. The degree to which differentiation and growth in vitro is similar to differentiation and growth in vivo requires rigorous testing, but the preliminary findings here suggest relevance of in vitro to in vivo cell development.

In summary, we observed reduced production of OLs in cells from subjects with SZ. We were able to look at correlations between cell characteristics in culture and related parameters in vivo in brain, but given the modest sample size, we had inadequate power to look for associations among cell measures, brain measures, and individual genes or gene sets. Such comparisons should become possible in time, but will likely require very large numbers of samples, as indicated by the modest associations observed between genes and illness. Of course, even at the cellular level, numerous interacting pathways probably determine risk. Nevertheless, OL development as a whole, not just particular pathways underlying OL development, may be tractable for modification in vivo. In this way, cell culture systems may be effective models for studies of the mechanisms underlying SZ and for drug discovery.
